# A Neural Controller Model Considering the Vestibulospinal Tract in Human Postural Control

**DOI:** 10.3389/fncom.2022.785099

**Published:** 2022-02-25

**Authors:** Yuichiro Omura, Kohei Kaminishi, Ryosuke Chiba, Kaoru Takakusaki, Jun Ota

**Affiliations:** ^1^Department of Precision Engineering, School of Engineering, The University of Tokyo, Tokyo, Japan; ^2^Research Into Artifacts, Center for Engineering, School of Engineering, The University of Tokyo, Tokyo, Japan; ^3^Division on Neuroscience, Department of Physiology, Asahikawa Medical University, Asahikawa, Japan

**Keywords:** postural control, forward dynamics simulation, neural controller model, musculoskeletal model, vestibulospinal tract

## Abstract

Humans are able to control their posture in their daily lives. It is important to understand how this is achieved in order to understand the mechanisms that lead to impaired postural control in various diseases. The descending tracts play an important role in controlling posture, particularly the reticulospinal and the vestibulospinal tracts (VST), and there is evidence that the latter is impaired in various diseases. However, the contribution of the VST to human postural control remains unclear, despite extensive research using neuroscientific methods. One reason for this is that the neuroscientific approach limits our understanding of the relationship between an array of sensory information and the muscle outputs. This limitation can be addressed by carrying out studies using computational models, where it is possible to make and validate hypotheses about postural control. However, previous computational models have not considered the VST. In this study, we present a neural controller model that mimics the VST, which was constructed on the basis of physiological data. The computational model is composed of a musculoskeletal model and a neural controller model. The musculoskeletal model had 18 degrees of freedom and 94 muscles, including those of the neck related to the function of the VST. We used an optimization method to adjust the control parameters for different conditions of muscle tone and with/without the VST. We examined the postural sway for each condition. The validity of the neural controller model was evaluated by comparing the modeled postural control with (1) experimental results in human subjects, and (2) the results of a previous study that used a computational model. It was found that the pattern of results was similar for both. This therefore validated the neural controller model, and we could present the neural controller model that mimics the VST.

## 1. Introduction

Postural control is essential for maintaining a stable standing posture, and thus it is a particularly important human motor function. Understanding human postural control is important in order to understand the disease mechanisms that lead to an impaired posture. This could lead to more effective rehabilitation strategies.

The basic neural mechanisms involved in standing upright are located in the brainstem and the spinal cord. The descending pathways from the brainstem to the spinal cord are responsible for the automatic processes that are involved in postural control (Takakusaki et al., 2017). Among these descending pathways, the reticulospinal tract (RST) and the vestibulospinal tract (VST) are particularly important for controlling posture (Takakusaki, 2017). The RST regulates muscle tone, the constant muscular tension that enables a standing posture to be maintained (Takakusaki et al., 1994, 2016). The RST both excites and inhibits muscles throughout the body (Magoun and Rhines, 1946; Rhines and Magoun, 1946). In contrast, the VST maintains the body and head in an upright position (Roberts, 1973; Schor, 1974; Takakusaki et al., 2017). It works by exciting the extensor muscles and inhibiting the flexor muscles, unlike the RST (Grillner et al., 1970; McCall et al., 2017).

It can be seen that both the VST and the RST are involved in maintaining an upright posture in humans. However, it has been suggested that damage to the VST underlies the impaired postural control seen in diseases such as Parkinson's disease and stroke (Miller et al., 2014; Lazzaro et al., 2018). This would suggest that the VST in particular plays an important role in controlling posture. Although neuroscientific methods have yielded a large amount of physiological data concerning the VST (Grillner et al., 1970; Roberts, 1973; Schor, 1974; McCall et al., 2017; Takakusaki et al., 2017), the exact contribution of the VST to postural control remains unclear. One reason for this is that the neuroscientific approach precisely verify input-output relationships under carefully controlled conditions. This can then limit our understanding of what happens when there is a wide array of sensory information and muscle outputs. To address this issue, computational models have been used to make and validate hypotheses about postural control.

Previous studies that modeled postural control have adopted an inverted pendulum model with 1~3 degrees of freedom (DOF) as a model of the human body (Kuo, 1995; Morasso et al., 1999; van der Kooij et al., 1999; Peterka, 2002; Peterka and Loughlin, 2004; Maurer and Peterka, 2005; Masani et al., 2006; Bottaro et al., 2008; Asai et al., 2009; Mahboobin et al., 2009). Neural controllers have been modeled with feedback (FB) control (Peterka, 2002; Mergner et al., 2003; Peterka and Loughlin, 2004; Mahboobin et al., 2009), as well as with both feedforward (FF) and FB control (Kuo, 1995; Morasso et al., 1999; van der Kooij et al., 1999). Neural controller models have also been developed that can compensate for the time delays involved in receiving sensory information (van der Kooij et al., 1999; Masani et al., 2006). For instance, a model developed by van der Kooij et al. integrates multisensory inputs and estimates posture using a Kalman filter, and was shown to maintain a standing posture with a time delay of 100 ms (van der Kooij et al., 1999). In another study, Masani et al. showed it was possible to compensate for a time delay by using FB control with sufficiently large FB gains (Masani et al., 2006). Intermittent control models have also been developed that use the mechanical properties of an inverted pendulum for intermittent control (Bottaro et al., 2008; Asai et al., 2009). Asai et al. compared a continuous FB controller with an intermittent controller. They showed that the intermittent controller was more robust than the continuous FB controller, because the model of the human body was stable with a smaller gain and with a larger parameter space (Asai et al., 2009).

A problem with the inverted pendulum model is that it simplifies the human anatomical structure to such an extent that it is difficult to see how it corresponds to the actual human skeleton and muscles. In addition, the inverted pendulum model controls the joint torque, which is the sum of the forces exerted by the muscles, rather than the activity of many muscles.

Computational modeling studies have also been conducted using musculoskeletal models, which have more DOF than the inverted pendulum model (Jiang et al., 2016; Kaminishi et al., 2019, 2020; Koelewijn and Ijspeert, 2020). These models better represent the human anatomical structure, and they include muscle control. Jiang et al. developed a musculoskeletal simulator that models human postural control. This was composed of a musculoskeletal model, with seven DOF and 70 muscles, and a neural controller model (Jiang et al., 2016) with proprioceptive FB control (from muscle length and lengthening velocity) and FF control from the RST. The simulator was able to maintain a standing posture with a time delay of up to 120 ms by modeling the muscle tone regulation mechanism. By introducing visual and vestibular FB control, it was possible to verify the relationship between sensory information and muscle tone (Jiang et al., 2017). The simulator has since been improved by Kaminishi et al. to investigate postural control when there is some disturbance (Kaminishi et al., 2019, 2020).

It can be seen that many studies have investigated postural control using computational models. As previously stated, human postural control is achieved by generating muscle output from sensory information *via* the descending tract. Furthermore, the relationship between descending tracts and postural control disorders in various diseases [Parkinson's disease (Lazzaro et al., 2018), stroke (Miller et al., 2014)] have been shown. Therefore, although the model considering both RST and VST is essential to examine postural control of these diseases, none of the studies using the inverted pendulum models considered the descending tracts. Besides, previous studies using the musculoskeletal model have only examined the importance of RST during the standing posture of healthy subjects.

This study aims to present a neural controller model that mimics the VST.

An overview of the method is given as follows. For this purpose, we constructed a neural controller mimicking the VST based on a previous model (Jiang et al., 2016). Furthermore, we constructed a musculoskeletal model having neck DOFs and muscles, which are needed to represent the VST. The validity of the model was evaluated by comparing the postural control seen in the model with (1) experimental results in human subjects using evaluation indices of postural sway (the velocity and the power spectral density (PSD) of postural sway), and (2) the results of a previous computational model, which showed that the effect of noise becomes smaller when muscle tone is greater (Kaminishi et al., 2020). The details are described in the next section.

## 2. Methods

### 2.1. Basic Design

#### 2.1.1. Task

This study focused on postural control when standing still because most people spend considerable time in this position. The task was to maintain an upright standing posture for 5,000 ms using the musculoskeletal model. This posture was chosen on the basis of a previous study (Kaminishi et al., 2019). As this model has many DOF and muscles, it is suitable for representing the function of the VST, which sends different signals to each muscle type and maintains the body in a vertical position. It was assumed that we could evaluate postural control by assessing the maintenance of a standing posture for 5,000 ms because we focused on static upright position.

#### 2.1.2. Assumption

We assumed the following:

1. Sensory information is only obtained from the vestibular and proprioceptive systems.
2. The contraction and relaxation of muscles with the same function (flexion and extension) in each joint are consistent with each other.
3. The control of each muscle is bilaterally symmetrical.

Concerning assumption 1, we considered the maintenance of an upright standing position when the eyes are closed. For this reason, the sensory information in the neural controller model was defined as vestibular and proprioceptive, excluding vision.

Concerning assumption 2, because the task was to maintain a static upright position, no postural change, such as taking a step, should occur; therefore, each joint movement was expected to be small. With such small joint movements, if the body falls forwards, the soleus and gastrocnemius muscles, which are the extensors of the ankle joint, would both contract. For this reason, we made assumption 2.

Concerning assumption 3, human muscles are bilaterally symmetrical, and because the task was to maintain a bilaterally symmetrical upright posture, we made assumption 3.

### 2.2. Computational Model

**Figure 1:**
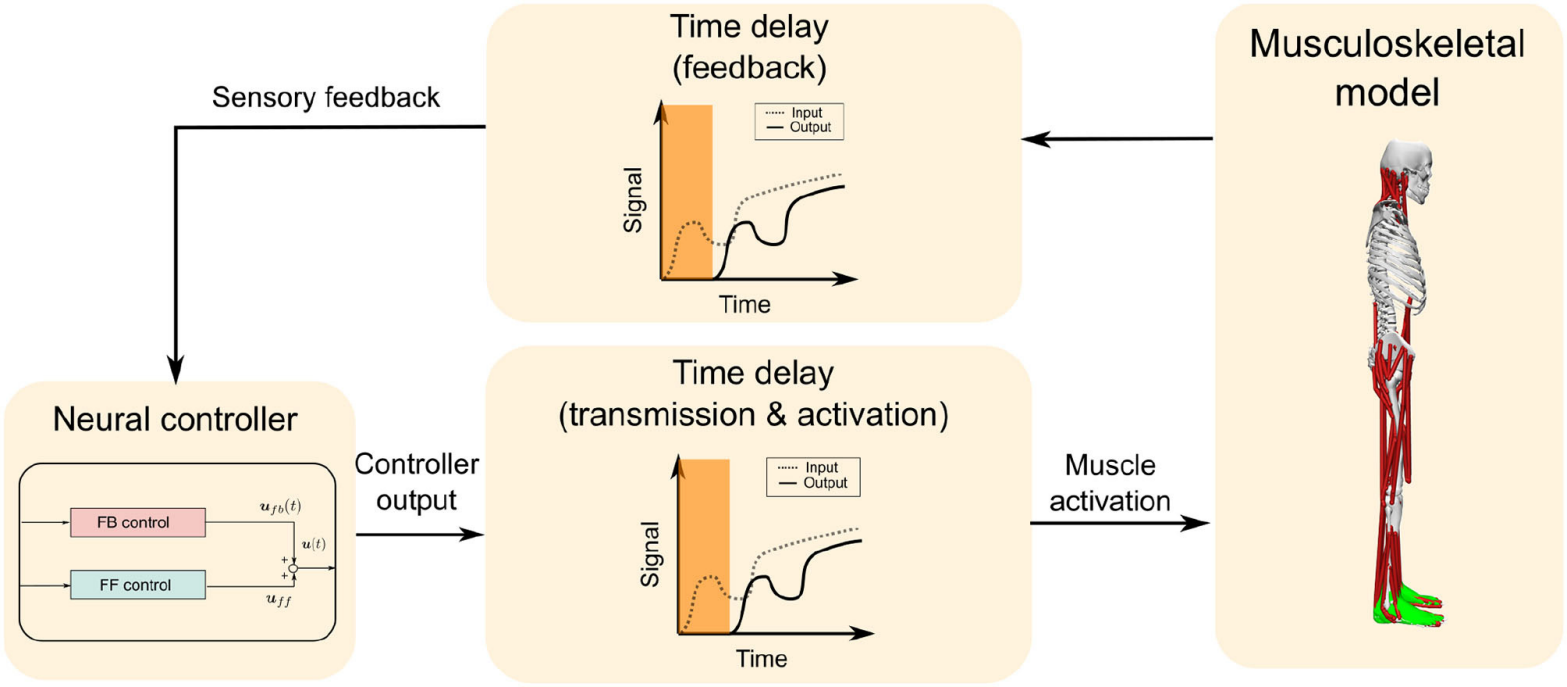
Overall design of the computational model. The computational model is composed of a musculoskeletal model and a neural controller model, which includes feedforward (FF) and feedback (FB) control. A time delay is added to the FB from the musculoskeletal model to the neural controller model, and also to the muscle activation output from the neural controller model to the musculoskeletal model, which totals 120 ms.

The computational model was composed of a musculoskeletal model that represents the human body and a neural controller model that controls it, as shown in Figure 1. The computational model was constructed and simulations were performed in OpenSim 4.0 SimTK.org (Delp et al., 2007; Seth et al., 2018) (Supplementary Materials Data Sheet 1).

#### 2.2.1. Musculoskeletal Model

**Figure 2:**
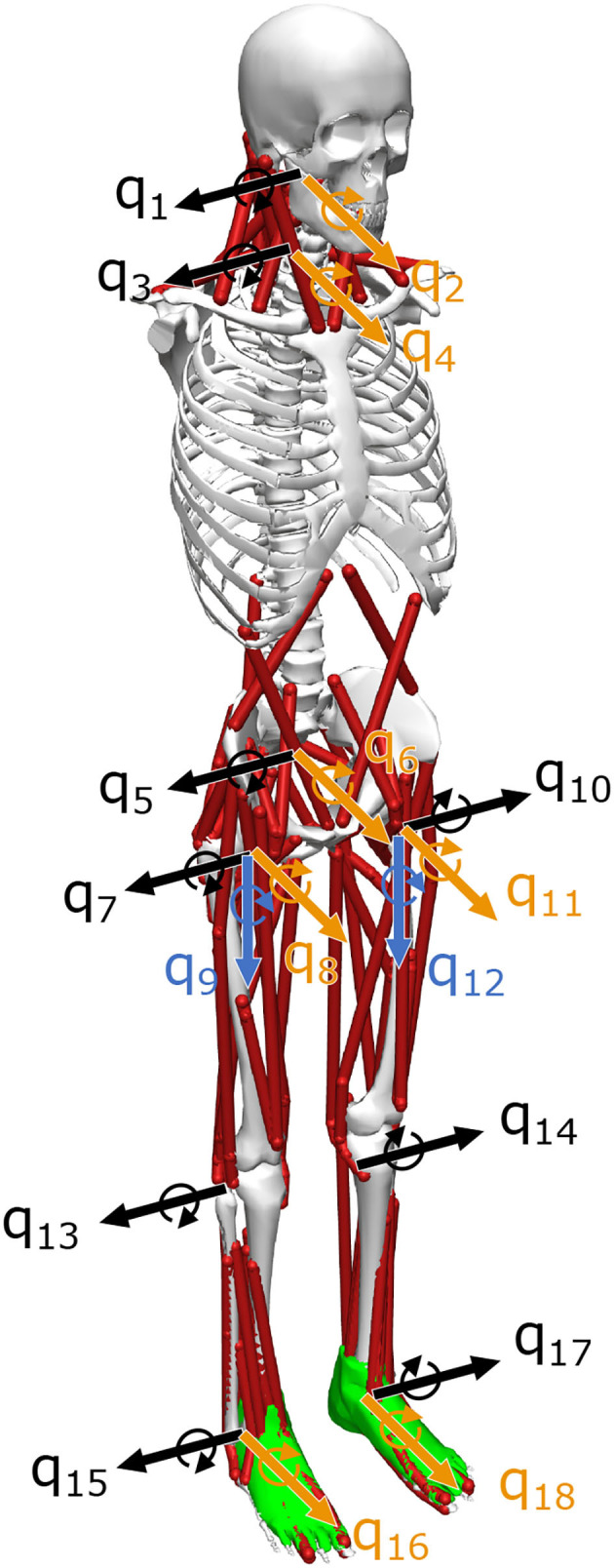
Musculoskeletal model. The musculoskeletal model has 94 muscles and 18 degrees of freedom (DOF). *q*_1_–*q*_18_ represent the DOF, where black is the sagittal plane, yellow is the frontal plane, and blue is the horizontal plane. The model is based on one developed in a previous study, but *q*_1_–*q*_4_ degrees of freedom have been added to the neck (Kaminishi et al., 2019).

The musculoskeletal model had 18 DOF and 94 muscles, including those of the neck related to the function of the VST (Figure 2). This model was based on the musculoskeletal model from a previous study, with four DOF (pitch, roll) added to the neck and 24 additional muscles to control these movements. The previous study had considered the upper part of the body to be a single rigid unit (Jiang et al., 2016, 2017; Kaminishi et al., 2019, 2020), and therefore these additional DOFs and muscles were necessary because our model included the vestibular sense, which is greatly influenced by head movements.

The center of rotation of the joint DOF and the skeletal and muscular parameters were based on the models developed by Vasavada et al. (1998), Thelen (2013), and Cazzola et al. (2017). The musculoskeletal model proposed by Cazzola et al. had 78 muscles in the neck. If we added all these muscles to the model, it becomes difficult to generate proper motion in the neck. The reason is that the calculation time to adjust control parameters increases exponentially as the number of muscles increases. The calculation to adjust control parameters is described in Section 2.2.3.2. Therefore, we added 24 neck muscles (see Supplementary Materials Table 3) to our model to reduce calculation time to adjust control parameters. The criteria were as follows. We excluded those muscles with a lower maximum isometric muscle force and a shorter length. However, to avoid large differences in the muscle force that can be exerted, the maximum isometric muscle force of the excluded muscles was added to that of the included muscles, which have the same function. The muscle model used was the Hill-type model (Millard et al., 2013).

#### 2.2.2. Neural Controller Model

**Figure 3:**
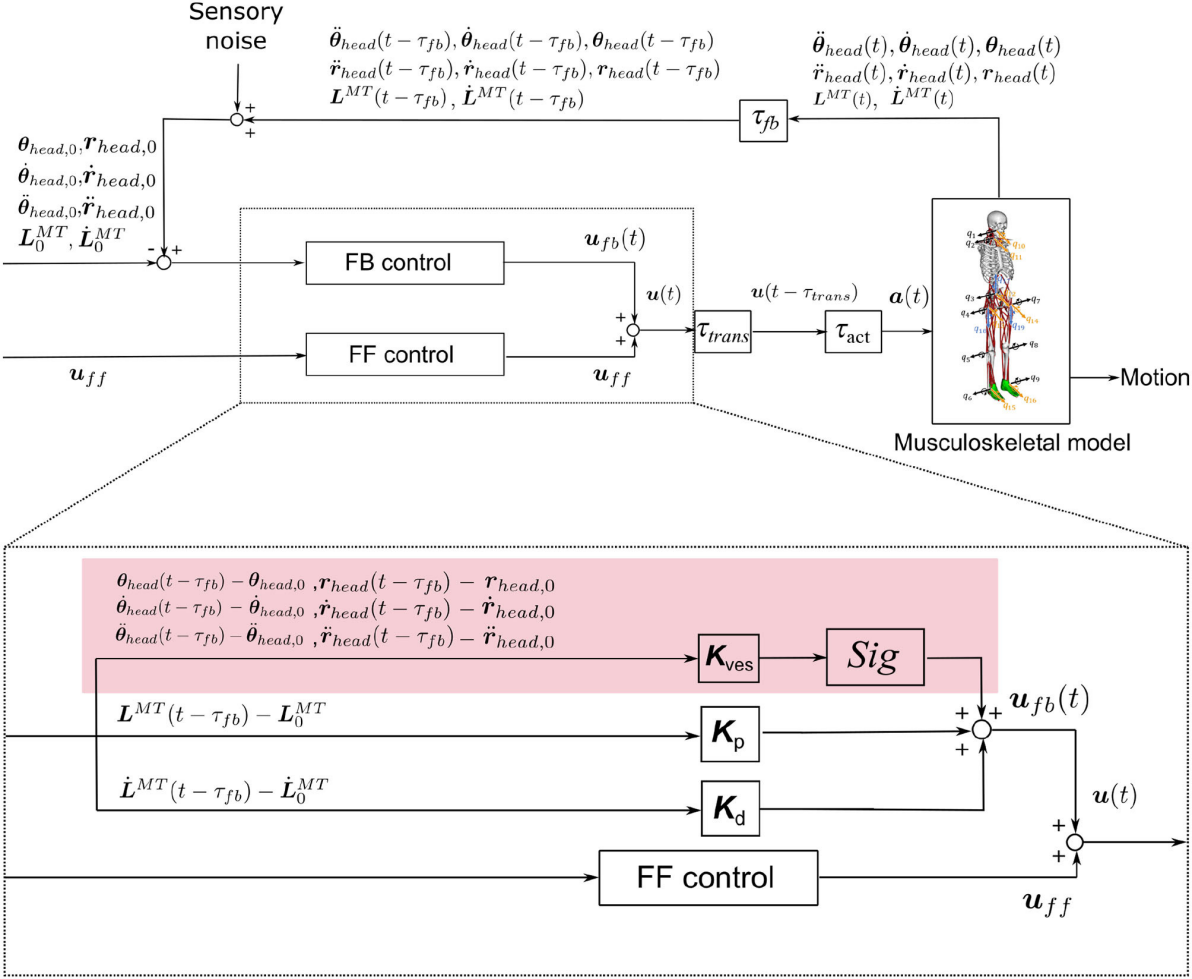
Neural controller model. The red area represents a control system that mimics the VST. ***K***_*p*_, ***K***_*d*_ are the FB gains. $L^{MT}\left(t-\tau_{\text{fb}}\right),{\dot{L}}^{MT}\left(t-\tau_{\text{fb}}\right)$ are the muscle length and the lengthening velocity, including the time delay associated with FB. ***K***_*ves*_ is the FB gain for the vestibular sensory information. τ_*fb*_, τ_*trans*_, and τ_*act*_ are time delays associated with FB, neurotransmission, and muscle activation/deactivation, respectively. ${\ddot{r}}_{x,head}$, ${\ddot{r}}_{y,head}$, and ${\ddot{r}}_{z,head}$ represent the translational acceleration of the head in the x, y, and z axes. ${\ddot{\theta }}_{x,head}$, ${\ddot{\theta }}_{y,head}$, and ${\ddot{\theta }}_{z,head}$ represent the angular acceleration of the head in the x, y, and z axes. ṙ_*x,head*_, ṙ_*y,head*_, and ${\ddot{r}}_{z,head}$ represent the translational velocities of the head in the x, y, and z axes. ${\dot{\theta }}_{x,head}$, ${\dot{\theta }}_{y,head}$, and ${\dot{\theta }}_{z,head}$ represent the head angular velocities in the x, y, and z axes. *r*_*x,head*_, *r*_*y,head*_, and *r*_*z,head*_ represent the head positions in the x, y, and z axes. θ_*x,head*_, θ_*y,head*_, and θ_*z,head*_ represent the head angles in the x, y, and z axes. *Sig* is a variable that changes the positive or negative value of the output. It is set to 1 if the output muscle is an extensor, and −1 if it is a flexor.

In this study, we considered both the VST and the RST. The neural controller model mimicked the VST, based on physiological data, and also included the RST proposed by Jiang et al. The proposed model is shown in Figure 3. It is composed of FF control, FB control, and time delays. The inputs are the initial values of the proprioceptive and vestibular information and also the values at *t* − τ_*fb*_ (time delays). The proprioceptive information is taken from the muscle lengths and the muscle lengthening velocities. The vestibular information is taken from the head acceleration, head angular acceleration, head velocity, head angular velocity, head position, and head angle. The outputs are the constant values for FF control (muscle tone, ***u***_*ff*_), which are determined through the RST, and the muscle activity of feedback control (***u***_*fb*_) that will correct the difference between the current posture and the target posture, which is determined using FB control. The summation of these outputs gives the resulting output from the neural controller (***u***). The ***u*** is converted into muscle activity ***a*** which involves a time delay and output to the musculoskeletal model. Forward dynamics simulations are performed with this muscle activity ***a***.

##### 2.2.2.1. FF Control

The FF control is based on previous studies. It models the muscle tone regulation carried out by the RST (Jiang et al., 2016). It is known that the RST controls muscle tone for the whole body, which involves constant muscular activity (Takakusaki et al., 2017). A certain amount of muscle tone is needed to maintain a posture and resist gravity.

The output from the FF control was therefore defined as muscle tone (***u***_*ff*_) in this study. It had a constant value because it involves continuous muscular activity that is not affected by time delays. The muscle tone could be set for each muscle, and various magnitudes of muscle tone could be set, even for the same posture. It was therefore possible to select multiple muscle tones for the same upright posture and to compare them. For this we used $||u_{\text{ff}}|{|}^{2}$, expressed in the following Equation (1), as the evaluation index of whole-body muscle tone to represent the muscle tone, which is a vector of 94 dimensions, with a scalar value (Jiang et al., 2016).

(1) $$\begin{array}{l} ||u_{\text{ff}}|{|}^{2}=\overset{n}{\underset{i=1}{\sum }}u_{ff,i}^{2} \end{array}$$

*u*_*ff,i*_ is the FF control output to the *i*th muscle. The *n* is the total number of muscles, which is *n* = 94 in this study. Similarly, we used $||u_{\text{fb}}|{|}^{2}$, expressed in the following Equation (2), as the evaluation index of muscle activity of feedback control.

(2) $$\begin{array}{l} ||u_{\text{fb}}|{|}^{2}=\overset{n}{\underset{i=1}{\sum }}u_{fb,i}^{2} \end{array}$$

##### 2.2.2.2. FB Control

FB control was included in the neural controller model to enable the difference between the target and the current posture to be corrected. It is known that humans use such FB control to maintain a standing posture. The target posture was set by specifying each joint angle. We adopted the upright standing position as our target posture, as in the previous study (Kaminishi et al., 2019). The initial posture was set to be the same as the target posture.

The FB control used vestibular sensory information, mimicking the VST, as described in Section 2.2.2.2.1, and also proprioceptive sensory information, as described in a previous study (Jiang et al., 2016).

*2.2.2.2.1. Physiology of the VST*. The VST can be divided into the lateral VST and the medial VST. The medial VST mainly connects to the cervical region (Perlmutter et al., 1998; McCall et al., 2017), and it has both excitatory and inhibitory effects on the cervical motoneurons (Wilson et al., 1970; Carleton and Carpenter, 1983; Takakusaki, 2017). It is involved in coordinating eye and neck movements (Takakusaki, 2017). The lateral VST extends along the entire spinal cord (Nyberg-Hansen and Mascitti, 1964), and it has an excitatory effect on extensor muscles and an inhibitory effect on flexor muscles (Grillner et al., 1970; McCall et al., 2017). It is involved in maintaining the head in a stable vertical position and in keeping the body vertical by using the vestibular sense (Roberts, 1973; Schor, 1974; Takakusaki et al., 2017). Because of the assumptions described in Section 2.1.2, only the lateral VST was included in the neural controller model; the medial VST was not included because it uses visual information.

*2.2.2.2.2. Vestibular FB Control Mimicking the VST*. We included FB control using vestibular information, which mimics the physiology of the VST, as described in Section 2.2.2.2.1. The vestibular information consisted of the head translational acceleration, head angular acceleration, head translational velocity, head angular velocity, head position, and head angle. This information is thought to be obtained in the vestibular organs and the vestibular nuclei. The following Equation (3)~(9) was used to express the output (*u*_*fb,ves,i*_) of FB control for the *i*th muscle using vestibular information.

(3) $$\begin{array}{l} u_{fb,ves,i}=sigK_{ves,i}e_{ves} \end{array}$$

(4) $$\begin{array}{l} sig=\{\begin{array}{ll} -1, & \text{Flexor muscles}\\ 1, & \text{Extensor muscles} \end{array} \end{array}$$

(5) $$\begin{array}{l} K_{ves,i}=\left[\begin{array}{c} K_{1,ves,i}K_{2.ves,i}\cdots K_{18,ves,i} \end{array}\right] \end{array}$$

(6) $$\begin{array}{l} e_{ves}=\left[\begin{array}{c} {\ddot{r}}_{head}\\ {\ddot{\theta }}_{head}\\ {\dot{r}}_{head}\\ {\dot{\theta }}_{head}\\ r_{head}\\ \theta_{head} \end{array}\right] \end{array}$$

(7) $$\begin{array}{l} r_{head}=\left[\begin{array}{c} \left|r_{x,head}-r_{x,head,0}\right|\\ r_{y,head}\\ \left|r_{z,head}-r_{z,head,0}\right| \end{array}\right] \end{array}$$

(8) $$\begin{array}{l} r_{head,y}=\{\begin{array}{ll} |r_{y,head}-r_{y,head,0}|, & \left(r_{y,head}-r_{y,head,0}<0\right)\\ 0, & \left(r_{y,head}-r_{y,head,0}\geq 0\right) \end{array} \end{array}$$

(9) $$\begin{array}{l} \theta_{head}=\left[\begin{array}{c} \left|\theta_{x,head}-\theta_{x,head,0}\right|\\ \left|\theta_{y,head}-\theta_{y,head,0}\right|\\ \left|\theta_{z,head}-\theta_{z,head,0}\right| \end{array}\right] \end{array}$$

*K*_1,*ves,i*_ ~ *K*_18,*ves,i*_ is the FB gain in the *i*th muscle based on vestibular information. ${\ddot{r}}_{x,head}$, ${\ddot{r}}_{y,head}$, ${\ddot{r}}_{z,head}$, ${\ddot{\theta }}_{x,head}$, ${\ddot{\theta }}_{y,head}$, ${\ddot{\theta }}_{z,head}$, ṙ_*x,head*_, ṙ_*y,head*_, ṙ_*z,head*_, ${\dot{\theta }}_{x,head}$, ${\dot{\theta }}_{y,head}$, ${\dot{\theta }}_{z,head}$, *r*_*x,head*_, *r*_*y,head*_, *r*_*z,head*_, θ_*x,head*_, θ_*y,head*_, and θ_*z,head*_ are the head translational acceleration, head angular acceleration, head translational velocity, head angular velocity, head position, and head angle for the x, y, and z axes, respectively. The subscript 0 for each variable represents the initial value. *sig* is −1 if the *i*th muscle is a flexor and 1 if it is an extensor. This causes the vestibular FB control to send inhibitory signals to flexor muscles and excitatory signals to extensor muscles (Equation 4). The VST keeps the body vertical, causing the head to move in the positive direction of the y-axis. This is expressed in the Equation (8). ***K***_*ves*_ ≠ *O* is defined as the presence of the VST. ***K***_*ves*_ = *O* is defined as the absence of the VST.

*2.2.2.2.3. Proprioceptive FB Control*. We included proprioceptive FB control, as in previous studies. The proprioceptive information was defined as the muscle-tendon's length and lengthening velocity. We considered that this information is obtained by converting the information from the muscle spindle and Golgi tendon organs. This is because the previous study has inferred that the length of the tendon is obtained from the Golgi tendon organ using a model (Kistemaker et al., 2013). The output (*u*_*fb,prop,i*_) of the proprioceptive FB control for the *i*th muscle is expressed in the following Equation (10) (Jiang et al., 2016):

(10) $$\begin{array}{l} u_{fb,prop,i}\left(t\right)=K_{p,i}\frac{L_{i}^{MT}\left(t-\tau_{fb}\right)-L_{i,0}^{MT}}{L_{i,0}^{MT}}+K_{d,i}\frac{{\dot{L}}_{i}^{MT}\left(t-\tau_{\text{fb}}\right)-{\dot{L}}_{i,0}^{MT}}{V_{i,max}} \end{array}$$

*K*_*p,i*_, *K*_*d,i*_ are the FB gains for the *i*th muscle based on the muscle length and the muscle lengthening velocity, respectively. $L_{i}^{MT}\left(t-\tau_{\text{fb}}\right)$, ${\dot{L}}_{i}^{MT}\left(t-\tau_{\text{fb}}\right)$ are the length and the lengthening velocity of the *i*th muscle, respectively, following the time delay that is associated with FB. $L_{i,0}^{MT}$ and ${\dot{L}}_{i,0}^{MT}$ are the initial values of the length and the lengthening velocity of the *i*th muscle, respectively. *V*_*i,max*_ is the maximum lengthening velocity of the *i*th muscle.

##### 2.2.2.3. Time Delays

There are various time delays in the human nervous system. We included three major time delays in our model (τ_*fb*_, τ_*trans*_, τ_*act, deact*_) (Jiang et al., 2016). τ_*fb*_ denotes the time delay for receiving sensory information from the sensory receptors. τ_*trans*_ denotes the time delay for sending signals to the muscles from the neural controller. τ_*act*_, τ_*deact*_ represents the time delay for either activating or deactivating the muscles. We set τ_*fb*_ = 40 ms, τ_*trans*_ = 40 ms, τ_*act*_ = 10 ms, τ_*deact*_ = 40 ms, in accordance with previous studies (Zajac, 1989; Winters, 1995; Masani et al., 2006).

##### 2.2.2.4. Sensory Noise

There is a certain amount of error in sensory information due to noise. This limits the ability to accurately detect the exact amount of body movement. In our model, we represented the noise in sensory information from the sensory receptors.

In previous studies, Gaussian noise added to the sensory information (van der Kooij et al., 2001; Peterka and Loughlin, 2004; Maurer and Peterka, 2005; Mahboobin et al., 2009). We also add Gaussian noise as sensory noise to each sensory information based on the previous studies. Let *y* be the sensory information used as feedback information by the neural controller model. *y* is given in the following Equation (11).

(11) $$\begin{array}{l} y=x+k_{noise}\mu X \end{array}$$

*x* is the true value of the sensory information. *k*_*noise*_ is a coefficient to change the magnitude of the noise. μ is the average of the absolute values for each sensory signal (*x*). We ran simulations in which the musculoskeletal model maintained an upright posture for 5,000 ms under the condition of *k*_*noise*_=0, and the average value of the sensory information of the standing results was used as μ. The noise level is determined by *k*_*noise*_ with μ as the standard noise level for each sensory signal. *X* is a computational random variable that follows a Gaussian distribution with a mean of zero and a standard deviation of one. We created *X* by using a random number generator. As it is difficult to measure the magnitude of human sensory noise (*k*_*noise*_) experimentally, the magnitude of the noise was set to *k*_*noise*_ = 0.01 in this study. The neural controller model generated muscle activity using *y*. Forward dynamics simulations were performed using this muscle activity. Deterministic differential equations were solved by the Runge-Kutta method using this muscle activity and the musculoskeletal model's state (Thelen et al., 2003).

#### 2.2.3. Control Parameter Adjustments

There were two control parameters in the neural controller model: the muscle tone (***u***_*ff*_) and FB gains (***K***_*p*_, ***K***_*d*_, ***K***_*ves*_). If these parameters are not adjusted, the musculoskeletal model cannot remain standing. Adjustments were therefore made, as described below.

The assumptions described in Section 2.1.2 meant that the FB gains of muscles belonging to the same functional group had the same value, and they were bilaterally symmetrical (note that muscle tone is independent of displacement; therefore, muscle tone can have a different value for each muscle). The muscles were divided into 14 groups according to the joint, whether they function in flexion or extension, and with reference to the moment arm generated by each muscle at the joint (Kaminishi et al., 2019) (Lumbar extensor, Lumbar flexor, Hip extensor, Hip flexor, Knee extensor, Knee flexor, Ankle extensor, Ankle flexor, Subtalar evertor, Subtalar invertor, Biarticular, Neck extensor, Neck flexor, Biarticular for neck). The parameters that could be adjusted were ***u***_*ff*_: 94 dimensions, ***K***_*p*_: 14 dimensions, ***K***_*d*_: 14 dimensions, and ***K***_*ves*_: 14 × 18 dimensions because there are 94 muscles in the musculoskeletal model.

**Figure 4:**
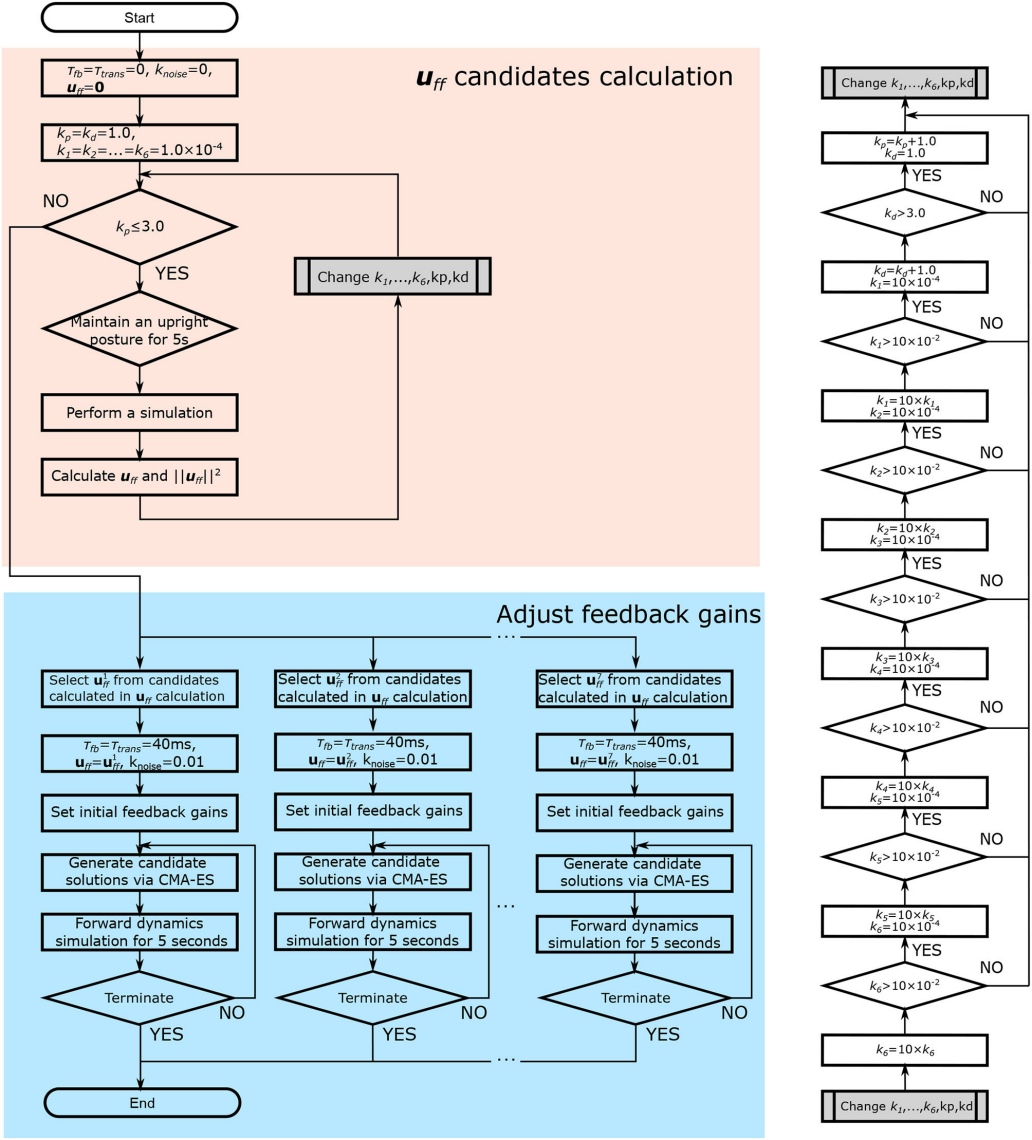
Flowchart of the parameter adjustment. First, muscle tone values (***u***_*ff*_) are calculated (shown in red). Muscle tones are selected, and the FB gain is adjusted using an optimization method (the Covariance Matrix Adaptation Evolution Strategy) (shown in blue). In the figure, τ_*fb*_ and τ_*trans*_ denote the time delay due to FB and neurotransmission, respectively. *k*_*noise*_ denotes the magnitude of the noise. *k*_*p*_ and *k*_*d*_ denote the FB gain for the proprioceptive information. *k*_1, …, 6_ denote the FB gain for the vestibular sense.

The muscle tone was not affected by time delay. In contrast, the FB gain was affected by the time delay. This was therefore adjusted with the time delay using an optimization method after calculating the muscle tone to efficiently adjust the control parameters (Figure 4). Previous studies using a similar model have shown that the muscle activity is closest to that of healthy subjects when the muscle tone is low (Jiang et al., 2016). On the basis of this, we calculated several muscle tone values and selected muscle tones including low muscle tone at regular intervals from calculated candidates. For each of these, we adjusted the FB gain to enable the musculoskeletal model to remain standing.

##### 2.2.3.1. Muscle Tone Calculation

Muscle tone is the continuous contraction of muscles that allows humans to remain standing. It therefore has a constant value that is independent of time delay. In our study, the muscle tone was calculated using the method described below. We set τ_*fb*_ = τ_*trans*_ = 0 ms (no time delay condition), *k*_*noise*_ = 0 (no noise condition), ***u***_*ff*_ = **0**, and various FB gains (based on Equations 14 ~ 16), and we ran the simulation to determine whether the musculoskeletal model could maintain a standing posture. If this was achieved, the muscle tone was calculated from the average muscle activity (Equation 12), using a method developed by Jiang et al. (2016).

(12) $$\begin{array}{l} u_{\text{ff,i}}=\frac{\int_{t1}^{t2}a_{i}\left(t\right)dt}{t_{2}-t_{1}} \end{array}$$

*u*_*ff,i*_ is the FF control output of the *i*th muscle. *t*_1_, *t*_2_ are set as *t*_1_ = 3,000 ms, *t*_2_ = 5,000 ms, in accordance with previous studies (Jiang et al., 2016). *a*_*i*_ is the muscle activity of the *i*th muscle. Similarly, the target joint angle was updated using the following Equation (13).

(13) $$\begin{array}{l} q_{i,target}=\frac{\int_{t1}^{t2}q_{i}\left(t\right)dt}{t_{2}-t_{1}} \end{array}$$

*q*_*i,target*_ is the target joint angle of the *i*th joint. *t*_1_, *t*_2_ are set as *t*_1_ = 3,000 ms, *t*_2_ = 5,000 ms. *q*_*i*_ is the joint angle of the *i*th joint.

By giving various FB gains as initial values, various muscle tones can be calculated. Thus, different FB gains were given to each muscle group by changing *k*_*p*_, *k*_*d*_, and ***k***_*ves*_, which are represented by the Equations (14) and (15), respectively. Depending on the combination of FB gains given, the musculoskeletal model may not be able to maintain a standing posture. We therefore calculated the muscle tones by giving various FB gains in the specified range. We were able to calculate various muscle tone candidates of multiple sizes because there were several FB gains that could maintain the standing position (see the upper side and the right side in Figure 4).


(14) $$\begin{array}{l} {}[K_{p,i},K_{d,i}]=\{\begin{array}{c} {}[0.50k_{p},0.23k_{d}],\text{ Lumbar extensor}\\ {}[0.48k_{p},0.11k_{d}],\text{ Lumbar flexor }\\ {}[0.45k_{p},0.05k_{d}],\text{ Hip extensor }\\ {}[0.50k_{p},0.16k_{d}],\text{ Hip flexor }\\ {}[0.33k_{p},0.05k_{d}],\text{ Knee extensor }\\ {}[0.28k_{p},0.23k_{d}],\text{ Knee flexor }\\ {}[0.17k_{p},0.06k_{d}],\text{ Ankle extensor }\\ {}[0.30k_{p},0.27k_{d}],\text{ Ankle flexor }\\ {}[0.50k_{p},0.11k_{d}],\text{ Subtalar evertor }\\ {}[0.50k_{p},0.05k_{d}],\text{ Subtalar inventor }\\ {}[0.39k_{p},0.05k_{d}],\text{ Biarticular }\\ {}[0.17k_{p},0.06k_{d}],\text{ Neck extensor }\\ {}[0.17k_{p},0.27k_{d}],\text{ Neck flexor }\\ {}[0.17k_{p},0.06k_{d}],\text{ Neck biarticular } \end{array} \end{array}$$

(15) $$\begin{array}{l} K_{ves,i}=0.01k_{ves} \end{array}$$

(16) $$\begin{array}{l} k_{ves}=\left[\begin{array}{cccccc} k_{1}e_{3} & k_{2}e_{3} & k_{3}e_{3} & k_{4}e_{3} & k_{5}e_{3} & k_{6}e_{3} \end{array}\right] \end{array}$$

***e***_3_ is a row vector of the third order with all of the elements being 1. The FB gains are empirically given in the range of 1.0 to 3.0 for *k*_*p*_ and *k*_*d*_, and in the range of 1.0 × 10^−4^ to 1.0 × 10^−2^ for *k*_1_ ~ *k*_6_. The coefficients were determined referring to the value in a previous study (Kaminishi et al., 2019).

In this study, seven muscle tones were selected from the candidates so that the evaluation index of muscle tone, $||u_{\text{ff}}|{|}^{2}$, was closest to values from 1.5 to 4.5 with an increment of 0.5. If the musculoskeletal model could not maintain a standing posture with selected uff in adjustment of feedback gains, the next closest uff was chosen. The reason for this was to compare postural control for a range of muscle tones including $||u_{\text{ff}}|{|}^{2}=2.0$, which was found, in a previous study, to be the closest to the muscle activity while standing still (Jiang et al., 2016), because there were too many calculated muscle tone candidates to perform the simulation with all of them.

##### 2.2.3.2. Adjustment of Feedback Gains

The musculoskeletal model cannot maintain the standing position using muscle tone alone when there are time delays. In real life, humans are thought to remain standing by using FB gains to adjust the muscle tone. The FB gains were therefore adapted so that the standing position could be maintained, even with time delays, using the optimization method.

The algorithm used for the adjustment was the Covariance Matrix Adaptation Evolution Strategy (CMA-ES) which is one of the evolution strategies (Hansen et al., 2003). This was chosen as it can be used for optimization with multimodality, thus making it suitable for tuning the FB gains in this study. In CMA-ES, forward dynamics simulations for 5,000 ms with time delays and noise are repeated to search for the FB gain that minimizes the objective function *J*, which is shown in the following Equations (17)~(20):

(17) $$\begin{array}{l} J=w_{\text{fail}}J_{\text{fail}}+w_{pos}J_{pos} \end{array}$$

(18) $$\begin{array}{l} J_{\text{fail}}=\frac{1}{T_{\text{fail}}}\left(T_{simu}-T_{\text{fail}}\right) \end{array}$$

(19) $$\begin{array}{l} T_{\text{fail}}=\{\begin{array}{ll} T_{stop}, & \left(h_{COM}<0.7m\right)\\ T_{simu}, & \left(h_{COM}\geq 0.7m\right) \end{array} \end{array}$$

(20) $$\begin{array}{l} J_{pos}=\overset{15}{\underset{j=1}{\sum }}\int_{0}^{T_{\text{fail}}}|\theta_{j}\left(t\right)-\theta_{j}\left(0\right)|dt \end{array}$$

*w*_*fail*_ is the weight of *J*_*fail*_, which evaluates the fall of the musculoskeletal model. *w*_*pos*_ is the weight of *J*_*pos*_, which evaluates the posture of the musculoskeletal model. *w*_*fail*_ and *w*_*pos*_ are set at 10,000 and 1, respectively, in accordance with previous studies (Kaminishi et al., 2019). The algorithm prioritizes the search for FB gains that do not cause the musculoskeletal model to fall. When this has been obtained, the algorithm then searches for the FB gain that minimizes *J*_*pos*_. In Equation (19), *T*_*simu*_ is the simulation time, which is 5,000 ms in this study. *T*_*stop*_ is the time at which the musculoskeletal model falls. Falling was defined as when the *h*_*COM*_ (the height of the center of mass (COM) of the musculoskeletal model) was below 0.7 m, or when the COM was out of the base of support (BOS). The threshold value of *h*_*COM*_ was set at 0.7 m because falling would be expected to occur at this point. For comparison, the *h*_*COM*_ of the musculoskeletal model is 0.72 m when the hip joint is rotated by 90 degrees; however, such a large joint flexion was not expected to occur because the upright standing position was maintained. When the COM is not within the BOS when standing upright, humans try to remain standing by stepping. We therefore regarded forward and backward deviations from the BOS as a fall because we focused on forward and backward swaying. θ_*j*_(*t*) is the joint angle of the *j*th joint at time *t*. *J*_*pos*_ is the time-integrated value of the change in the joint angle from the initial position.

These parameters were not adjusted to fit the experimental data because we adjusted them using the optimization method with an objective function that evaluates the fall and posture of the musculoskeletal model.

### 2.3. Evaluation

This section describes how the validity of the neural controller model was evaluated. Simulations were run to determine whether the musculoskeletal model would remain standing for 5,000 ms, using the adjusted control parameters described in Section 2.2.3. This included various conditions of muscle tone and with the VST either present or absent. The evaluation index was calculated from the simulation. To consider the effect of noise, we performed ten simulations for each condition. The noise was changed in each simulation because the random seed was changed. The following three evaluation indices were used for the validity assessment:

- The center of pressure (COP) velocity
- The slope of the PSD of COP
- The correlation coefficient between the standard deviation of the COP velocity and the muscle tone

These evaluation indices were chosen on the basis of findings reported in previous studies. For instance, it has been found that patients with vestibular disorders have a significantly greater COP velocity compared with healthy subjects (Talebi et al., 2016; Sprenger et al., 2017). Furthermore, differences have been found for the slope of the PSD of COP between patients with vestibular disorders and healthy subjects. Specifically, the slope β_1_ in the ~1 Hz region has been found to be larger in the positive direction, and the slope β_2_ in the 1~5 Hz region has been found to be larger in the negative direction for patients (Aoki et al., 2014). The increase in β_1_ in the positive direction indicates that the power tends to be lower at lower frequencies and higher at higher frequencies in the ~1 Hz region, resulting in finer fluctuations. The increase in β_2_ in the negative direction indicates that the power tends to be higher at lower frequencies and lower at higher frequencies in the 1~5 Hz region. In other words, this indicates that the postural sway contains more low-frequency components in the 1~5 Hz region. In another study, computational models showed that the effect of sensory noise decreases as the muscle tone increases (Kaminishi et al., 2020). This means that the variation (standard deviation) in COP velocity due to noise should decrease as the muscle tone increases. These previous studies led us focus on the three evaluation indices listed above.

In our model, the conditions with and without the VST represented healthy subjects and patients with vestibular disorders, respectively. The COP velocity was calculated in the anterior-posterior direction (the average of 10 trials), because the VST was considered to play a large role in this direction. The COP velocity in each condition were compared between the conditions with and without the VST using *t*-test. *P* < 0.0083 was considered statistically significant with a Bonferroni correction. The PSD of COP was calculated and then β_1_ and β_2_ were determined. Because the sampling frequency of the simulation was not constant, the Lomb-Scargle method was used to calculate the PSD of COP. This was then normalized to sum to 1. The slopes in the ~1 Hz area (β_1_) and the 1~5 Hz area (β_2_) were calculated using linear regression in log-log plot (Aoki et al., 2014). The PSD was calculated for the trial with the median objective function calculated from Equation (17), where the effect of noise was considered to be intermediate among the ten trials.

## 3. Results

Several muscle tone (FF output) candidates were calculated using the method described in Section 2.2.3.1. We could calculate 1,442 muscle tone candidates for the condition with the VST and 383 muscle tone candidates for the condition without the VST. Seven of these were selected in the vicinity of $||u_{\text{ff}}|{|}^{2}=1.5,\;2.0,\;2.5,\;3.0,\;3.5,\;4.0,\;4.5$, at even intervals. The exact values of these were $||u_{\text{ff}}|{|}^{2}=1.50,1.93,2.48,3.00,3.55,3.95,4.45$ for the condition with the VST, and $||u_{\text{ff}}|{|}^{2}=2.09,2.56,2.97,3.56,3.94,4.51$ for the condition without the VST (Supplementary Materials Table 1, Data Sheet 2). For the former condition (with the VST), it was possible to calculate muscle tone values of approximately $||u_{\text{ff}}|{|}^{2}=1.5$, whereas this was not the case for the latter condition (without the VST); here, a muscle tone below $||u_{\text{ff}}|{|}^{2}=1.97$ could not be calculated. In other words, without the VST, the musculoskeletal model could not maintain a muscle tone lower than $||u_{\text{ff}}|{|}^{2}=1.97$, even without a time delay. The FB gain was adjusted using the method described in Section 2.2.3.2 for each selected muscle tone (Supplementary Materials Table 2). Using the optimization method, it was possible to determine the FB gain that would enable the musculoskeletal model to remain standing for 5,000 ms. This was the case for all of the selected muscle tones.

**Figure 5:**
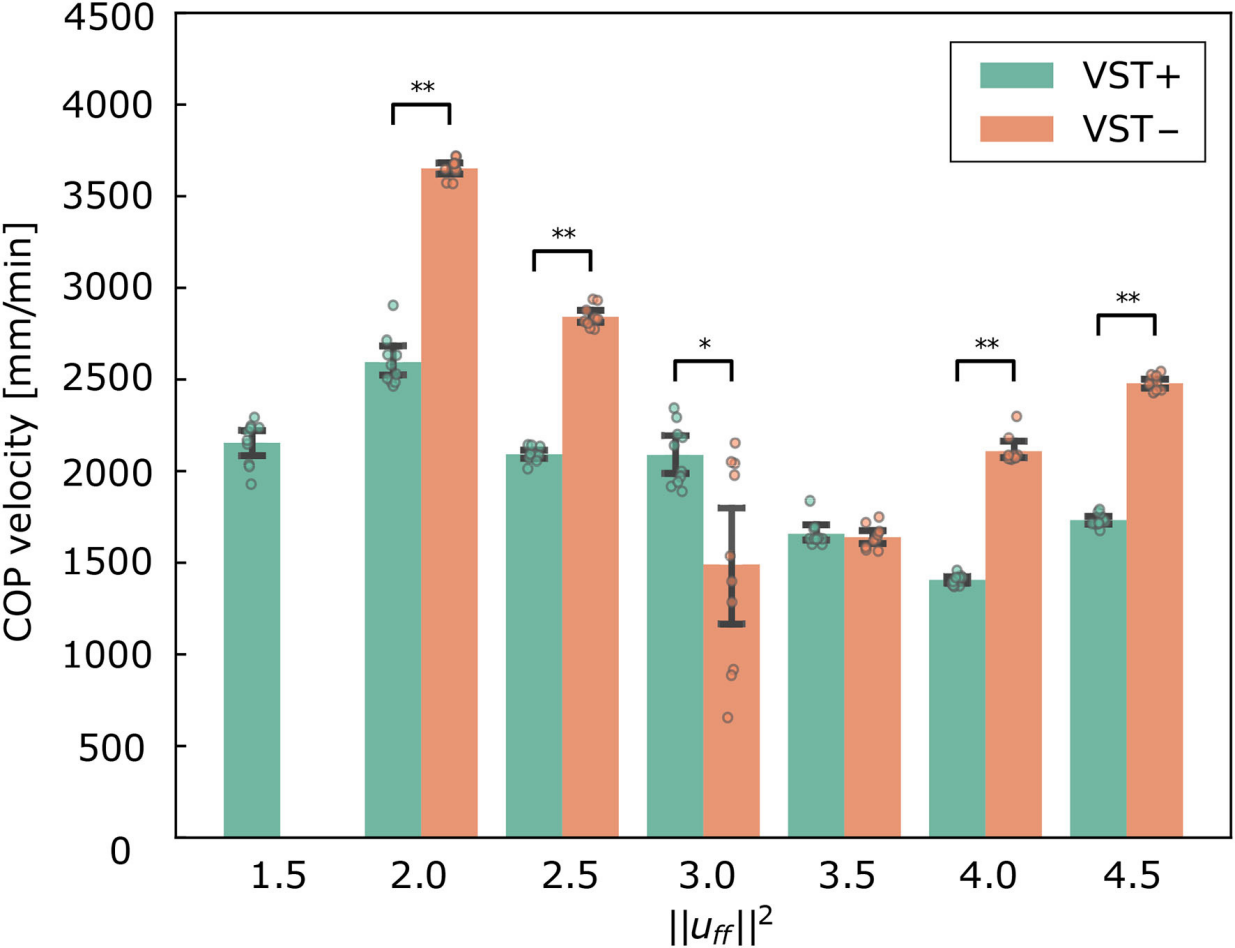
Center of pressure (COP) velocity in the forward and backward directions for each condition. Mean ± S.D. are plotted on the graph. VST ± denotes the presence or absence of the vestibulospinal tract (VST). For VST-, the value is 0 where the muscle tone $||u_{\text{ff}}|{|}^{2}=1.5$ could not been calculated. *P < 0.050. **P < 0.010.

Figure 5 shows the COP velocities with and without the VST for each muscle tone (Supplementary Materials Data Sheet 3). The COP velocities were found to be significantly lower with the VST for all of the muscle tones except $||u_{\text{ff}}|{|}^{2}=3.0,\;3.5$ (*P* < 0.0010). The COP velocities were significantly lower without the VST for $||u_{\text{ff}}|{|}^{2}=3.0$ (*P* = 0.0076), and were not different for $||u_{\text{ff}}|{|}^{2}=3.5$. The lowest COP velocity was for $||u_{\text{ff}}|{|}^{2}=3.0$ without the VST and for $||u_{\text{ff}}|{|}^{2}=4.0$ with the VST.

**Figure 6:**
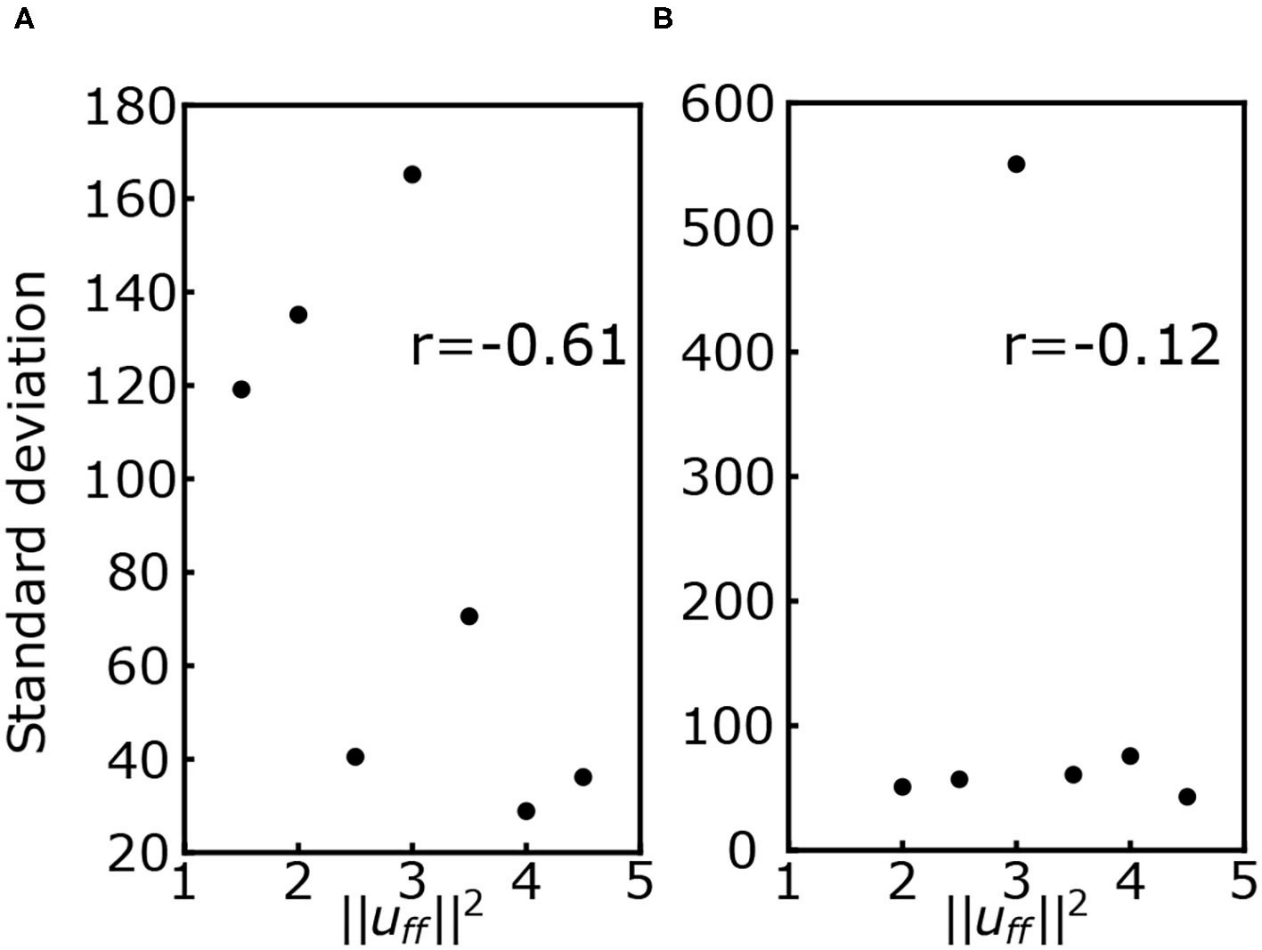
**(A)** Standard deviation of the center of pressure (COP) velocity for each muscle tone with the VST. **(B)** Standard deviation of the COP velocity for each muscle tone without the VST. r denotes the correlation coefficient.

Figure 6 shows the standard deviation of the COP velocity for each muscle tone in conditions with and without the VST. The correlation coefficient between $||u_{\text{ff}}|{|}^{2}$ and the standard deviation of the COP velocity was *r* = −0.61 for the condition with the VST, and *r* = −0.12 for the condition without the VST.

**Figure 7:**
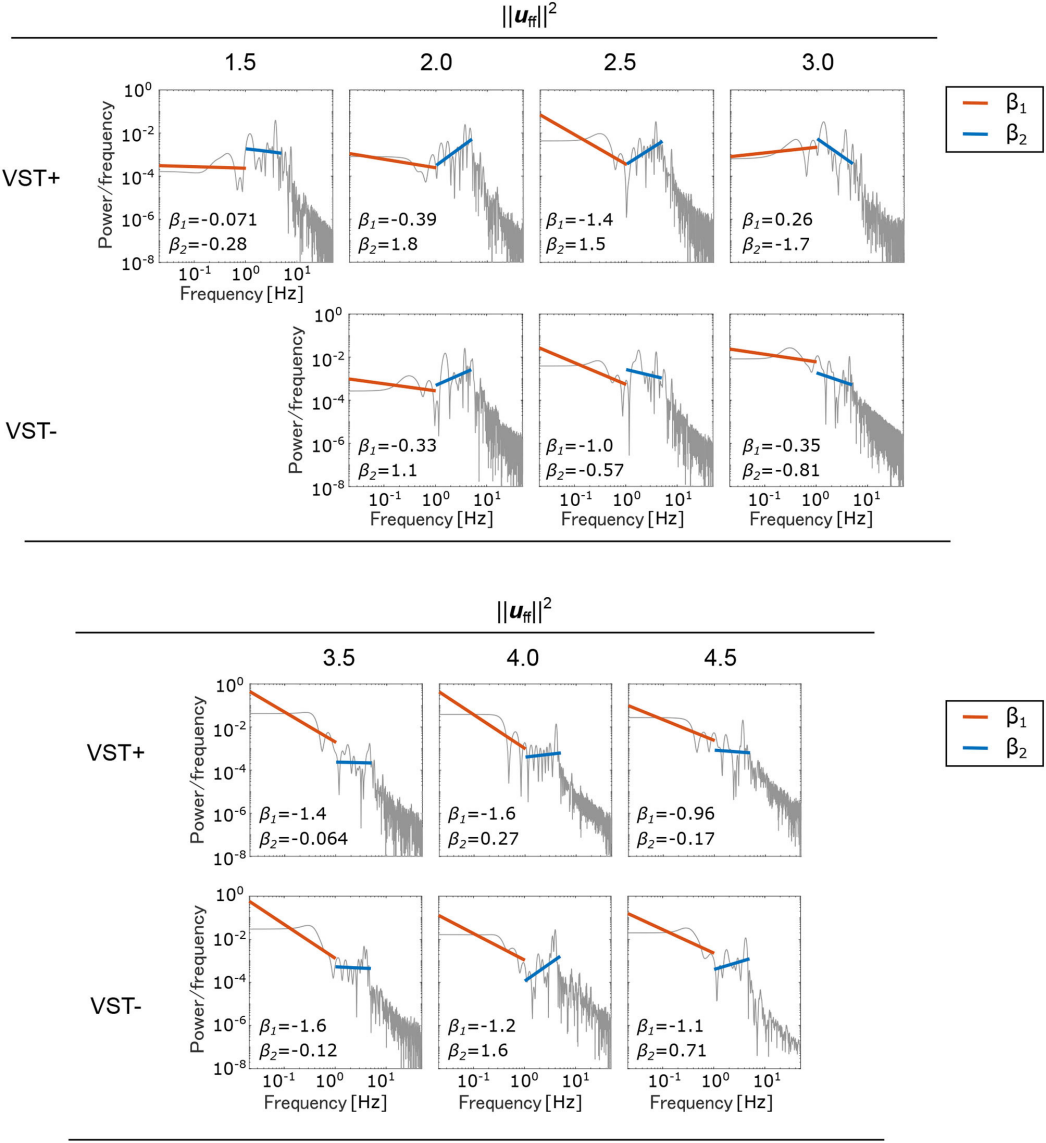
Power spectral density (PSD) of the center of pressure (COP). A separate log-log plot is shown for each condition. The PSDs for the seven selected muscle tones are shown; VST ± denotes the presence or absence of the VST. For VST-, a muscle tone could not be calculated where $||u_{\text{ff}}|{|}^{2}=1.5$; the corresponding field is therefore blank. The PSDs were normalized to sum to 1. The slope β_1_ in the low-frequency region (~1 Hz) and the slope β_2_ in the high-frequency region (1~5 Hz) are both shown on the graphs.

**Figure 8:**
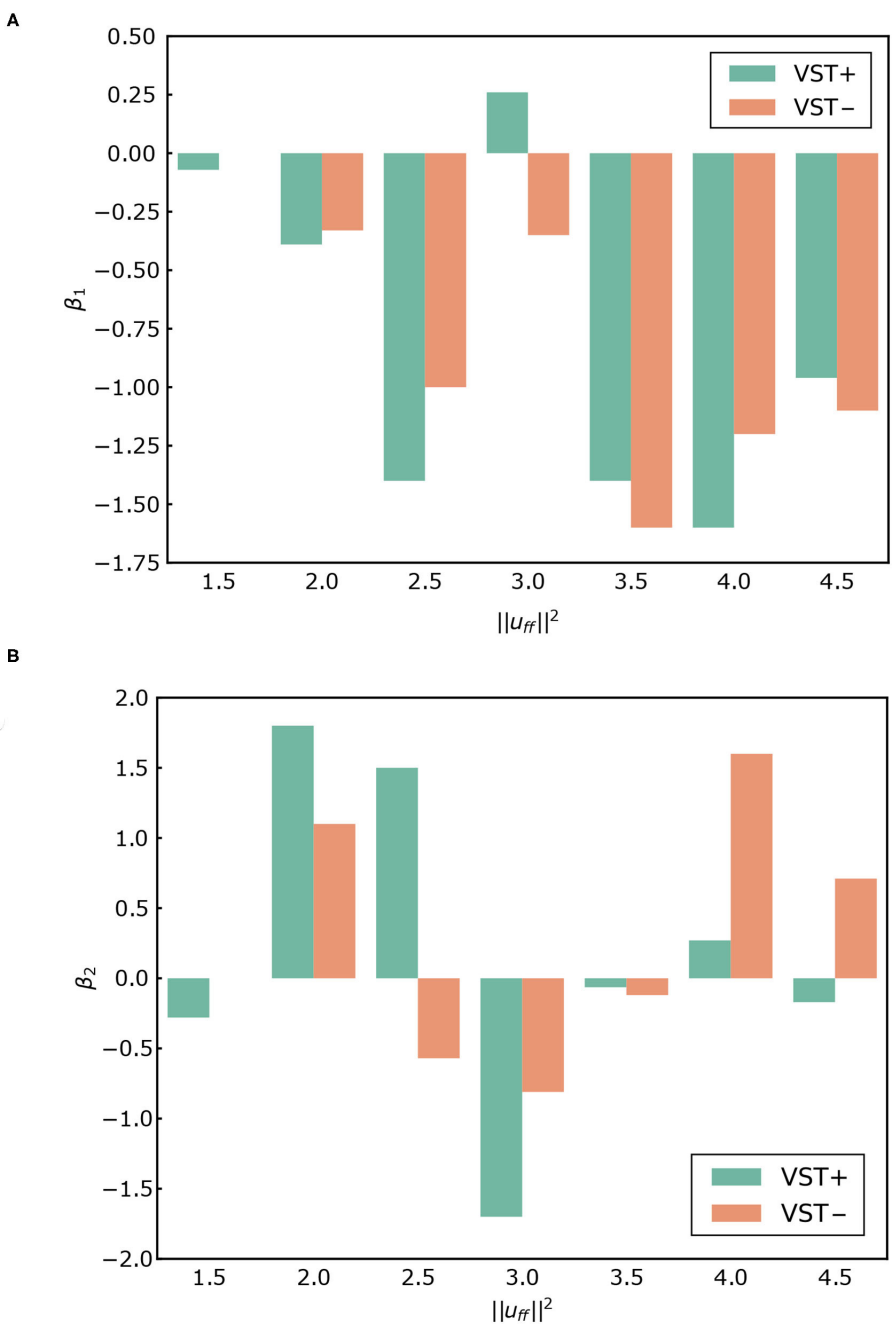
**(A)** β_1_ for each condition. **(B)** β_2_ for each condition. VST ± denotes the presence or absence of the vestibulospinal tract (VST). For VST-, the value is 0 where the muscle tone $||u_{\text{ff}}|{|}^{2}=1.5$ could not been calculated.

Figure 7 shows the PSD and the slopes β_1_, β_2_. Figure 8 shows the slopes β_1_, β_2_. For $||u_{\text{ff}}|{|}^{2}=1.5\sim 2.5$, β_1_ was larger in the negative direction, and β_2_ was larger in the positive direction with the VST. For $||u_{\text{ff}}|{|}^{2}=3.0\sim 4.5$, β_1_ tended to be larger in the negative direction, and β_2_ tended to be larger in the positive direction without the VST.

**Figure 9:**
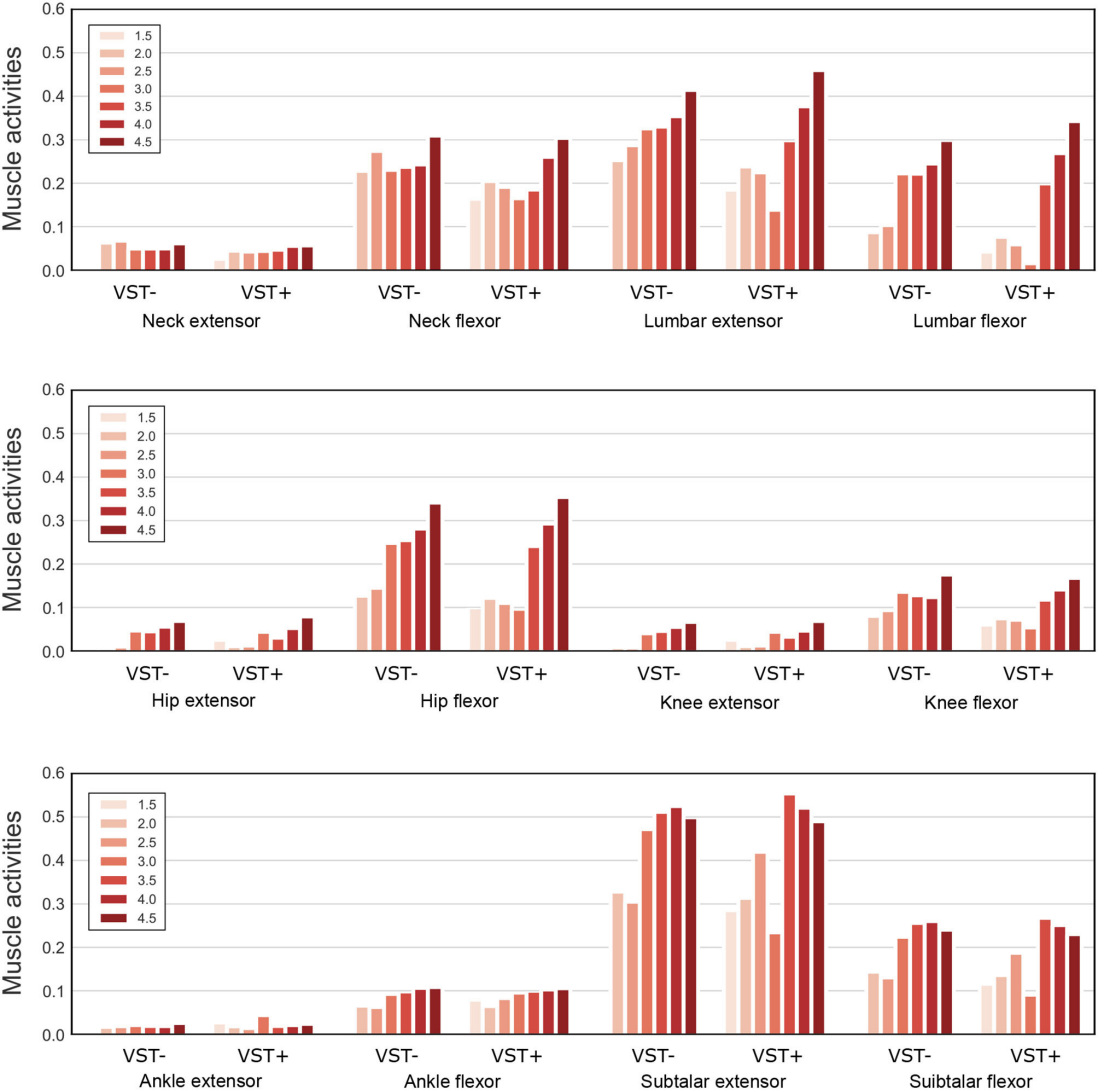
Muscle tone of the extensor and flexor muscles at each joint. Muscle tones for seven values of $||u_{\text{ff}}|{|}^{2}$ are plotted on the graph. For VST-, the value is 0 where the muscle tone $||u_{\text{ff}}|{|}^{2}=1.5$ could not been calculated. VST ± denotes the presence or absence of the VST.

Figure 9 shows the muscle tone for each muscle at the selected $||u_{\text{ff}}|{|}^{2}$.

**Figure 10:**
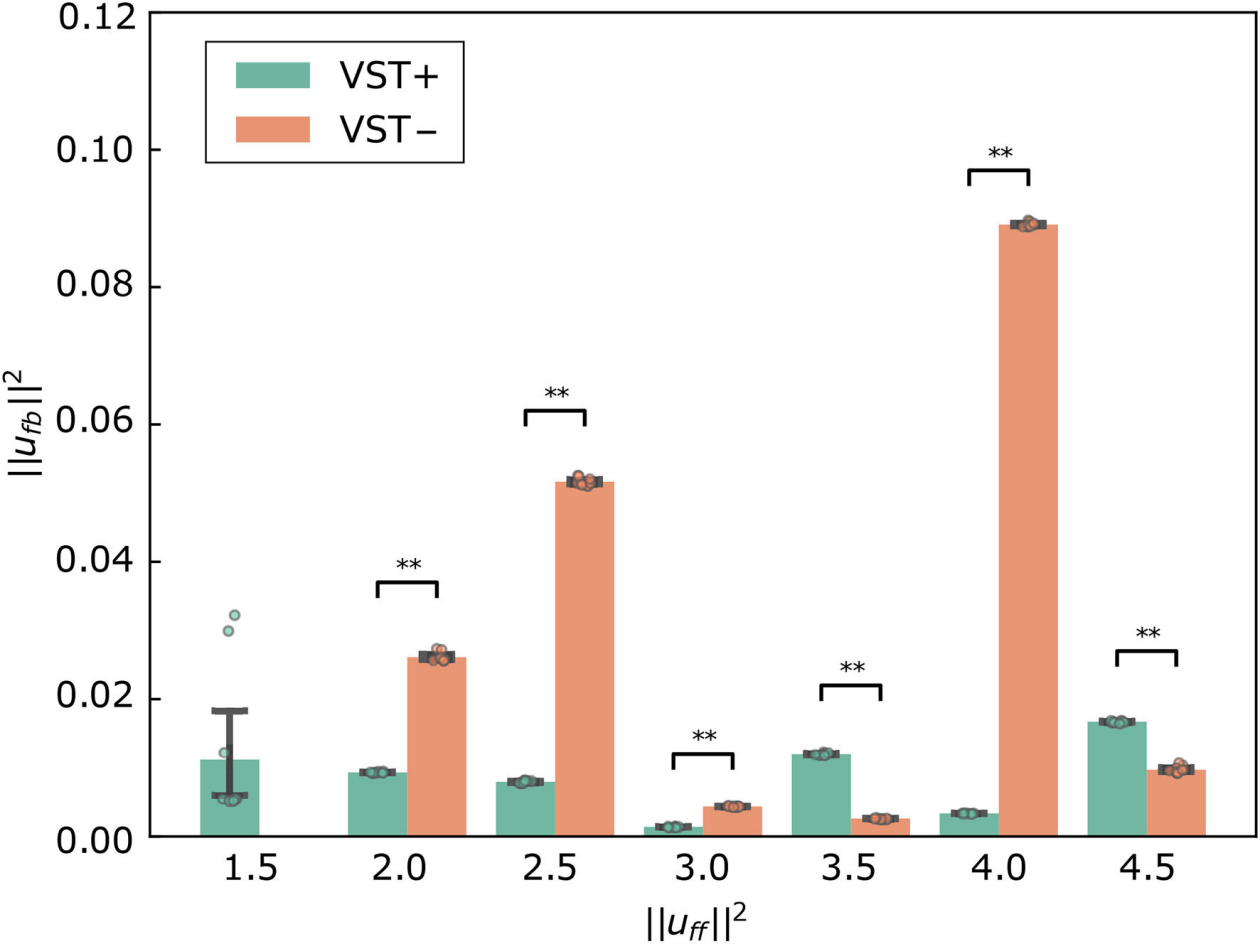
$||u_{\text{fb}}|{|}^{2}$ for each condition. Mean ± S.D. are plotted on the graph. VST ± denotes the presence or absence of the vestibulospinal tract (VST). For VST-, the value is 0 where the muscle tone $||u_{\text{ff}}|{|}^{2}=1.5$ could not been calculated. **P < 0.010.

Figure 10 shows $||u_{\text{fb}}|{|}^{2}$ for each condition. The value of $||u_{\text{fb}}|{|}^{2}$ was significantly lower in the condition with the VST than in the condition without the VST for $||u_{\text{fb}}|{|}^{2}=1.5\sim 3.0$ (*P* < 0.0010). The value of $||u_{\text{fb}}|{|}^{2}$ was significantly higher in the condition with the VST than in the condition without the VST for $||u_{\text{fb}}|{|}^{2}=3.5\sim 4.5$, except for $||u_{\text{fb}}|{|}^{2}=4.0$ (*P* < 0.0010). For $||u_{\text{fb}}|{|}^{2}=4.0$, the value of $||u_{\text{fb}}|{|}^{2}$ was significantly lower in the condition with the VST than in the condition without the VST (*P* < 0.0010).

Table 1 shows the means of the joint angle during simulation and target value of joint angle in a sagittal plane corresponding to the swaying in the anterior-posterior direction (*q*_1_, *q*_3_, *q*_5_, *q*_7_, *q*_13_, *q*_15_ in Figure 2). The data of all joint angles are included in the Supplementary Materials Table 4.

**Table 1:** Joint angle.

**Conditions**	** $||u_{ff}|{|}^{2}$ **	**Source**	**Joint (°)**					
			***q***_**1**_	***q***_**3**_	***q***_**5**_	***q***_**7**_	***q***_**13**_	***q***_**15**_
VST+	1.5	Mean	2.9 ± 0.088	−0.14 ± 0.13	−11 ± 0.095	−5.8 ± 0.062	2.0 ± 0.011	2.1 ± 0.053
		Target	2.8	0.053	−11	−5.9	1.9	2.1
	2.0	Mean	2.3 ± 0.26	−0.25 ± 0.16	−11 ± 0.069	−5.9 ± 0.073	2.0 ± 0.011	2.4 ± 0.046
		Target	1.8	0.0081	−11	5.8	1.7	2.3
	2.5	Mean	2.2 ± 0.19	−0.21 ± 0.20	−12 ± 0.13	−5.9 ± 0.069	2.0 ± 0.011	2.3 ± 0.035
		Target	1.8	−0.017	−11	−5.8	1.9	2.2
	3.0	Mean	10 ± 0.11	0.71 ± 0.31	−11 ± 0.17	−5.9 ± 0.10	2.1 ± 0.011	1.9 ± 0.053
		Target	9.7	0.92	−11	−6.0	2.0	1.8
	3.5	Mean	2.8 ± 0.84	0.61 ± 0.69	−12 ± 0.31	−5.4 ± 0.098	1.9 ± 0.025	2.2 ± 0.092
		Target	1.4	0.045	−11	−5.5	1.5	2.2
	4.0	Mean	2.5 ± 0.22	0.085 ± 0.16	−11 ± 0.081	−5.4 ± 0.044	1.8 ± 0.033	2.1 ± 0.048
		Target	2.1	0.11	−11	−5.4	1.5	2.2
	4.5	Mean	3.8 ± 0.29	0.0055 ± 0.21	−11 ± 0.15	−5.4 ± 0.051	1.9 ± 0.031	2.1 ± 0.050
		Target	2.6	0.18	−11	−5.4	1.5	2.1
VST-	2.0	Mean	3.1 ± 0.21	−0.35 ± 0.16	−12 ± 0.12	−5.9 ± 0.10	2.0 ± 0.016	2.4 ± 0.058
		Target	1.8	−0.032	−11	−5.7	1.7	2.3
	2.5	Mean	3.8 ± 0.44	−0.48 ± 0.29	−11 ± 0.20	−5.9 ± 0.12	1.9 ± 0.017	2.5 ± 0.067
		Target	2.0	0.021	−11	−5.8	1.7	2.3
	3.0	Mean	2.0 ± 0.65	−0.19 ± 0.97	−12 ± 0.12	−5.4 ± 0.059	1.9 ± 0.017	2.3 ± 0.030
		Target	1.4	0.020	−11	−5.4	1.6	2.2
	3.5	Mean	1.9 ± 0.36	−2.6 ± 0.65	−11 ± 0.13	−5.4 ± 0.10	1.9 ± 0.032	2.1 ± 0.077
		Target	1.9	0.081	−11	−5.4	1.6	2.2
	4.0	Mean	2.5 ± 0.29	−1.7 ± 0.21	−11 ± 0.089	−5.3 ± 0.056	1.9 ± 0.020	2.0 ± 0.050
		Target	2.0	0.13	−11	−5.3	1.7	2.1
	4.5	Mean	2.9 ± 0.24	0.058 ± 0.25	−12 ± 0.14	−5.3 ± 0.060	1.9 ± 0.046	2.2 ± 0.084
		Target	2.3	0.18	−11	−5.4	1.5	2.2

*The means of joint angle during simulation and target value of joint angle in sagittal plane corresponding to the swaying in the anterior-posterior direction (q_1_, q_3_, q_5_, q_7_, q_13_, q_15_ in Figure 2) for each conditions. The lower limbs are partially omitted due to symmetry. VST ± denotes the presence or absence of the vestibulospinal tract*.

## 4. Discussion

### 4.1. Validation of the Neural Controller Model

#### 4.1.1. COP Velocity

Figure 5 shows that the COP velocities were significantly larger without the VST for all of the selected muscle tones, with the exception of $||u_{\text{ff}}|{|}^{2}=3.0,\;3.5$. This result is consistent with experimental results in human subjects (Talebi et al., 2016; Sprenger et al., 2017).

The results for $||u_{\text{ff}}|{|}^{2}=1.5,\;2.0$ show that, with the VST, the musculoskeletal model could maintain a standing posture with a lower muscle tone and a lower COP velocity than without the VST. This indicates that the VST enabled the musculoskeletal model to maintain a standing posture when the muscle tone was low. In line with this, a previous study reported that muscle tone increases when sensory information is inhibited during standing (Chiba et al., 2013). It is consistent with this study that the presence or absence of the VST caused a difference in muscle tone to remain standing.

Concerning the relationship between the COP velocity and the muscle tone, it could be seen that the COP velocity decreased as the muscle tone increased. The COP velocity was lowest at $||u_{\text{ff}}|{|}^{2}=4.0$ in the condition with the VST, and at $||u_{\text{ff}}|{|}^{2}=3.0$ in the condition without the VST. Above these values, the COP velocity increased as the muscle tone increased. It is conceivable that when the muscle tone is less than $||u_{\text{ff}}|{|}^{2}=3.0,\;4.0$, the muscle stiffness increases as the muscle tone increases. This would decrease the magnitude of the postural sway and thus reduce the COP velocity. However, when the muscle tone is greater than $||u_{\text{ff}}|{|}^{2}=3.0,\;4.0$, the increased stiffness could cause an increase in high frequency oscillations, which would then increase the COP velocity. In line with this, Figure 7 shows that the power of frequencies approximately 3~5 Hz is greater for muscle tones above $||u_{\text{ff}}|{|}^{2}=3.0$.

Figure 5 shows that the COP velocities were significantly smaller without the VST for $||u_{\text{ff}}|{|}^{2}=3.0$ and not different between with and without the VST for $||u_{\text{ff}}|{|}^{2}=3.5$. Several feedback gains were larger in the condition without the VST at $||u_{\text{ff}}|{|}^{2}=3.0$. The large feedback gains reduce the displacement. However they are usually not selected by the optimization method because the musculoskeletal model may become unstable depending on the noise with large feedback gains. It is considered that the large feedback gains were selected at $||u_{\text{ff}}|{|}^{2}=3.0$ because $||u_{\text{ff}}|{|}^{2}=3.0$ was the value that allowed the model to maintain a stable standing posture. COP velocity was therefore very small for some noises. We think that the musculoskeletal model was unstable with the possibility of falling due to the large feedback gains at $||u_{\text{ff}}|{|}^{2}=3.0$ when the number of trials and the noise increased. Regarding $||u_{\text{ff}}|{|}^{2}=3.5$, the COP velocity was not different between the conditions with and without the VST. Additionally, the muscle tone at which the smallest COP velocity was achieved was different for each condition, due to the difference in the controller. Considering this, when COP velocity was compared between different muscle tones, there was a difference between the condition without the VST at $||u_{\text{ff}}|{|}^{2}=3.5$ and the condition with the VST at $||u_{\text{ff}}|{|}^{2}=4.0$. We therefore concluded that the musculoskeletal model can maintain a more stable standing posture in the condition with the VST.

#### 4.1.2. Slope of the PSD of COP

It has previously been reported that the slope of the PSD of COP in the ~1 Hz region, β_1_, is larger in the positive direction in patients with vestibular disorders than in healthy subjects. The mean of β_1_ was reported to be -1.22 for healthy subjects and -1.02 for patients with vestibular disorders (Aoki et al., 2014). In our study, the value of β_1_ was larger in the positive direction in the condition without the VST than in the condition with the VST for $||u_{\text{ff}}|{|}^{2}=1.5\sim 2.5$, as shown in Figures 7, 8. This indicates that the amplitude of the higher frequency components increases in the ~1 Hz region when there is no VST, i.e., the postural sway becomes finer. This difference between the presence and absence of the VST is consistent with the studies on patients with vestibular disorders. It was noted that the β_1_ at $||u_{\text{ff}}|{|}^{2}=2.5$ was closest to that found in a study comparing patients with healthy controls. This implies that humans may maintain a standing posture using a muscle tone of approximately $||u_{\text{ff}}|{|}^{2}=2.5$. This would be in line with previous findings (Jiang et al., 2016).

In contrast to this, β_1_ was found to be larger in the negative direction in the condition without the VST for the higher muscle tones ($||u_{\text{ff}}|{|}^{2}=3.0\sim 4.5$), with the exception of $||u_{\text{ff}}|{|}^{2}=4.0$. This result is not consistent with the experimental results obtained in human subjects. This may be because the muscle tone is higher than that found in healthy individuals ($||u_{\text{ff}}|{|}^{2}=2.0$). Such an increase in muscle tone would increase the stiffness, which would reduce the amount of FB control needed to maintain an upright posture. In this way, the effect of the VST would not be apparent.

It has previously been reported that the slope β_2_ in the 1~5 Hz region is larger in the negative direction in patients with vestibular disorders compared with that of healthy controls (Aoki et al., 2014). In our study, β_2_ increased in the negative direction in the condition without the VST at low muscle tones ($||u_{\text{ff}}|{|}^{2}=1.5\sim 2.5$), as shown in Figures 7, 8. This is consistent with the patients' results in that, for both, the power of the lower frequencies tends to increase, and the power of the higher frequencies tends to decrease in the 1~5 Hz region. However, in the patient study, β_2_ was negative, whereas in the computational model, β_2_ was found to be positive for many of the conditions. This may be due to the high sampling frequency in the computational model (approximately 1,000 Hz in this study). In one previous study, the PSD of COP was compared for conditions with intermittent control and continuous control using an inverted pendulum model. It was found that the high-frequency component increased when there was continuous control, whereas the low-frequency component increased when there was intermittent control (Asai et al., 2009). Although it is difficult to investigate the time intervals for control in humans, the sampling frequency is considered to have a significant effect on the PSD of COP. In our study, β_1_ at $||u_{\text{ff}}|{|}^{2}=2.5$ was close to the results found in humans. However, by controlling at a lower sampling frequency, it is possible to obtain β_1_ and β_2_ values that are closer to those found in humans, even when the muscle tone is lower.

For the higher muscle tones ($||u_{\text{ff}}|{|}^{2}=3.0\sim 4.5$), β_2_ was found to be larger in the positive direction without the VST, with the exception of $||u_{\text{ff}}|{|}^{2}=3.5$. This is not in line with the results found in patients, and may be due to the fact that the muscle tones are higher than those found in healthy individuals, as considered for β_1_. It is possible that the effect of the VST was not apparent at these higher muscle tones because of the increase in stiffness.

#### 4.1.3. Correlation Between the Standard Deviation of the COP Velocity and the Muscle Tone

A previous study using a musculoskeletal model found that the effect of noise decreases as the muscle tone increases (Kaminishi et al., 2020). In line with this, we found a negative correlation between the muscle tone and the standard deviation of the COP velocity for the condition with the VST, as shown in Figure 6A. This result indicates that the effect of noise becomes smaller as the muscle tone increases. It is possible that this is due to an increase in joint stiffness at the higher muscle tones, which would lead to a smaller effect of noise. This would be consistent with the previous computational model.

For the condition without the VST, we found that there was no correlation between the muscle tone and the standard deviation of the COP velocity, as shown in Figure 6B. This may relate to the fact that with the VST, FB information is obtained from both vestibular and proprioceptive senses, with an effect of noise added to both. In contrast, without the VST, only proprioceptive information is used, and thus the effect of the noise is smaller. Because greater muscle tone is associated with smaller effect of noise, the muscle tone here may have been sufficiently large for the magnitude of given noise without the VST. As a result, the standard deviation of the COP velocity would be smaller without the VST than with the VST, and no correlation would be observed.

### 4.2. Muscle Tones at Which the Musculoskeletal Model Can Remain Standing

For the condition with the VST, we were able to obtain a muscle tone corresponding to $||u_{\text{ff}}|{|}^{2}=1.5$; without the VST, this was not achieved. In other words, the musculoskeletal model could not remain standing without the VST at a muscle tone equivalent to $||u_{\text{ff}}|{|}^{2}=1.5$, even without time delays. This indicates that the muscle tone at which the musculoskeletal model can remain standing depends on whether the VST is functioning. This result is consistent with previous studies (Chiba et al., 2013).

### 4.3. Change of Muscle Activity of FB Control by the VST

The comparison of ***u***_*fb*_ may not be appropriate as an evaluation index of the controller because ***u***_*fb*_ is affected by small oscillations that do not affect the stability; however a certain trend can be observed. The value of $||u_{\text{fb}}|{|}^{2}$ was significantly lower in the condition with the VST than in the condition without the VST for $||u_{\text{ff}}|{|}^{2}=1.5\sim 3.0$, as shown in Figure 10. This indicates that the VST enables the musculoskeletal model to maintain a standing posture with low ***u***_*fb*_ for low muscle tone. The value of $||u_{\text{fb}}|{|}^{2}$ was significantly higher in the condition with the VST than in the condition without the VST for $||u_{\text{ff}}|{|}^{2}=3.5\sim 4.5$. In the presence of sufficiently large muscle tone to stabilize, the condition with the VST is expected to have a greater ***u***_*fb*_ than the condition without the VST because of the difference in muscle activity between flexors and extensors.

At $||u_{\text{ff}}|{|}^{2}=1.5$, the vestibular feedback gains were large. These gains may have been larger to maintain a standing posture with lower muscle tone. It is considered that the variation was large at $||u_{\text{ff}}|{|}^{2}=1.5$ because of these large vestibular feedback gains. The value of $||u_{\text{fb}}|{|}^{2}$ was significantly large at $||u_{\text{ff}}|{|}^{2}=4.0$ without the VST because of a small oscillation of the neck, as mentioned above. With high muscle tone, oscillations occur depending on the patterns of muscle tone.

### 4.4. Effects of Sensory Noise

In this study, the magnitude of the noise was set at *k*_*noise*_ = 0.01 in Equation (11). The musculoskeletal model could remain standing at all of the selected muscle tones with a time delay, regardless of the VST, and this suggests that there was not enough noise to cause a fall. In line with this, it is known that there is noise in human sensory information, but this does not cause healthy people to fall. We therefore concluded that the magnitude of the noise in this study was not excessive.

In our study, we found that the standard deviation of the COP velocity was higher at $||u_{\text{ff}}|{|}^{2}=3.0$ with the VST (Figures 5, 6). This result indicates that the effect of noise increases at this muscle tone. The reason for this can be determined by examining (Figure 9). This shows that the muscle tone of the ankle extensors increased, while the muscle tone of the lumbar, knee, and hip joint muscles decreased at $||u_{\text{ff}}|{|}^{2}=3.0$ compared with the other muscle tones. The increased muscle tone in the ankle extensors would lead to a reduced COP velocity. However, the stiffness of each joint would have decreased because of a reduced co-contraction magnitude. As a result, the effect of noise would have increased.

In addition, we found that the standard deviation of the COP velocity was larger at $||u_{\text{ff}}|{|}^{2}=3.0$ without the VST than that at other $||u_{\text{ff}}|{|}^{2}$ values (Figures 5, 6). As mentioned in Section 4.1.1, several feedback gains were larger in the condition without the VST at $||u_{\text{ff}}|{|}^{2}=3.0$ than at other $||u_{\text{ff}}|{|}^{2}$ values. Noise may reverse the number sign of sensory information. This can lead to an increase or decrease in muscle activity that is contrary to what is needed. The large feedback gains magnify this effect. The condition without the VST at $||u_{\text{ff}}|{|}^{2}=3.0$ would therefore have large variation.

### 4.5. Strength of Proposed Model

We proposed a neural controller model and a musculoskeletal model. Regarding the neural controller model, we proposed a model that mimics the VST. We believe that this neural controller model can be used to validate posture control focusing on the VST. By varying model parameters, it would be possible to simulate the prediction of symptoms and make detailed proposals for rehabilitation. Moreover, since both the RST and VST were modeled, it would be possible to examine the effects and interactions of each the descending tract on postural control when disease or injury impair these descending pathways.

Alternatively, regarding the musculoskeletal model, we proposed a model having additional degrees of freedom and neck muscles to the model from the previous study. Although the proposed model increased computational cost, we could model the function of the VST. Furthermore, Table 1 shows that the model could maintain a posture close to the target posture with a difference of fewer than about three degrees. This result suggests that the proposed musculoskeletal model which added the neck joints and muscles could represent the stable motion of the neck during static standing. We concluded that this musculoskeletal model is therefore enough to simulate static standing. Using the proposed multi-degree of freedom musculoskeletal model, it is possible to investigate individual muscle activities and coordinated movements of polyarticular muscles, compared to previous models with fewer degrees of freedom, such as the inverted pendulum model. Additionally, the proposed model, including the neck, can express the abnormal posture seen in Parkinson's disease.

### 4.6. Limitations

The proposed musculoskeletal model does not include arms. Instead, their mass was added to the torso. However, it is known that the arms contribute to postural stability, especially in difficult standing positions, such as tandem standing (standing with one leg in front of the other) and standing on one leg. It has previously been found that a difficult standing position is more stable when the arms are free, while a normal standing position is more stable when the arms are fixed (Objero et al., 2019). If we were to add arms to our musculoskeletal model, we would be able to examine their effect on postural control under various conditions.

Muscles of the musculoskeletal model did not consider short-range stiffness. Muscle short-range stiffness is a muscle property that increases muscle force in muscle fiber stretch. This property is essential in the stability of a body with perturbations (De Groote et al., 2017). This property was not considered in this study because we focused on static standing positions. However, it should be considered when dynamic posture control is focused on in the future.

In this study, FB information was obtained only from the vestibular system and proprioception because we assumed that the eyes were closed. We considered the maintenance of an upright position with closed eyes to focus on the vestibular system. In fact, many tests of standing posture control are conducted with closed eyes. Visual information is crucial when postural control is difficult, such as during external disturbances or when standing on foam. It is therefore necessary in the case of evaluating difficult postural control conditions. Hence, we hope to be able to add visual feedback to this model in the future.

The proposed neural controller model did not consider estimations through the cerebellum. The reason for this was because we believed that the cerebellum's contributions of estimation and learning were smaller during static and stable standing than dynamic and challenging standing (Foerster et al., 2017). However, it has been reported that the cerebellum is more active in the static standing than in the supine position (Ouchi et al., 1999), thus we may need to consider the cerebellum in the future.

The proposed neural controller also did not consider stretch reflex control and cognitive effects. However, we consider that the model was enough because we could validate this model concerning the focused phenomenon (the effect of the VST on static standing). A limitation of modeling studies is that it is difficult to create a model that includes all elements. This is because the more elements are included, the more difficult it becomes to correspond each element to the results. There is also a limit to the number of parameters. The computational cost is high even in this research. The simulation took about 100 h for each condition using 30 threads (Intel Xeon, 36 cores, 2.60 GHz) and simulations for 13 conditions took about 1,300 h in total. As the number of parameters is increased, the computational cost increases exponentially. Therefore, we aim to construct a model that can explain the phenomenon with as few elements as possible.

## 5. Conclusions and Future Perspectives

This study presents a neural controller model that mimics the VST, which was constructed on the basis of physiological findings. The model's validity was evaluated by reproducing human postural control using adjusted parameters. The results were compared with findings from human subjects and previous computational models, and revealed similar trends for both. The study involved adjusting various parameters using the objective function which evaluates whether the musculoskeletal model can remains standing. In other words, the parameters are not adjusted to match the experimental results in humans. Nevertheless, as in the experiments in human subjects, there was a significant difference in COP velocity of the proposed model. In addition, the slope of the PSD of COP in the ~1 Hz region was larger in the positive direction in the condition without the VST than in the condition with the VST and the slope of the PSD of COP in the 1~5 Hz region was larger in the negative direction in the condition without the VST than in the condition with the VST. These results were also consistent with those of human experiments. We therefore conclude that the validity of the neural controller model was confirmed, and could present the neural controller that mimics the VST. Our future work will focus on disorders of the descending tract and how these affect patients' motor control. We will use our computational model to better understand the mechanisms involved and to improve our understanding of postural control disorders, such as Parkinson's disease.

## Data Availability Statement

The original contributions presented in the study are included in the article/Supplementary Material, further inquiries can be directed to the corresponding author.

## Author Contributions

YO performed the simulations, analyzed the data, and wrote the manuscript. KK helped to perform the simulations. KK, RC, KT, and JO provided a review of the research methods. JO supervised the study. All authors designed the study, discussed the results, and reviewed the article. All authors contributed to the article and approved the submitted version.

## Funding

This work was supported by JSPS KAKENHI, Grant-in-Aid for Scientific Research on Innovative Areas Hyper-adaptability for overcoming body-brain dysfunction: Integrated empirical and system theoretical approaches (Grant Numbers 19H05722 and 19H05730), and the Mohammed bin Salman Center for Future Science and Technology for Saudi-Japan Vision 2030 at the University of Tokyo (MbSC2030).

## Conflict of Interest

The authors declare that the research was conducted in the absence of any commercial or financial relationships that could be construed as a potential conflict of interest.

## Publisher's Note

All claims expressed in this article are solely those of the authors and do not necessarily represent those of their affiliated organizations, or those of the publisher, the editors and the reviewers. Any product that may be evaluated in this article, or claim that may be made by its manufacturer, is not guaranteed or endorsed by the publisher.
